# Peripheral Hematological and Immune-Endocrine Markers in Children with Specific Learning Disorder: An Exploratory Retrospective Case–Control Study

**DOI:** 10.3390/brainsci16080776

**Published:** 2026-07-23

**Authors:** Dilek Altun Varmış, Cumali Yüksekkaya, Hülya Binokay, Serkan Güneş, Nazmiye İnce, Elif Koçak, Elif Gözde Yüce Antepüzümü, Kübra Şahin, Esra Erol Tanrıkulu

**Affiliations:** 1 Department of Child and Adolescent Psychiatry, University of Health Sciences, Adana City Training and Research Hospital, 01060 Adana, Türkiye; drcumaliyuksekkaya@gmail.com (C.Y.); drnazmiyeocalan@gmail.com (N.İ.);; 2 Department of Biostatistics, Çukurova University, 01330 Adana, Türkiye; hulyabinokay@gmail.com

**Keywords:** specific learning disorder, peripheral biomarkers, neuropsychological assessment, molecules to cognition, Wechsler Intelligence Scale for Children—Revised, cognitive function, translational neuroscience, systemic inflammation, mean platelet volume

## Abstract

**Highlights:**

**What are the main findings?**

- Children with specific learning disorders showed a selective peripheral hematological pattern, mainly lower mean platelet volume and higher eosinophil count after false discovery rate correction.
- Complete blood count-derived inflammatory indices showed weak discriminative validity and no corrected association with global intelligence quotient or selected cognitive domains.

**What are the implications of the main findings?**

- Routine hematological indices should not be used as screening or diagnostic biomarkers for specific learning disorder.
- The findings support prospective, standardized molecules-to-cognition studies integrating direct immune measures, clinical comparison groups, and contemporary cognitive and academic assessments.

**Abstract:**

Background: Specific learning disorder (SLD), termed “specific learning disability” in the Human Phenotype Ontology (HP:0001328), is a heterogeneous neurodevelopmental disorder, and the association between peripheral biological changes and cognitive function remains not well understood. Peripheral hematological and immune-endocrine biomarkers and their relationships with neuropsychological evaluations were evaluated in children with SLD. Methods: In this retrospective case–control study, we evaluated the peripheral biomarkers of a complete blood count and biochemical panel in 103 children with SLD and 102 well-child outpatient controls. Within the SLD group, we examined the association of systemic inflammatory indices with cognitive domains as assessed by the Wechsler Intelligence Scale for Children—Revised. Results: Group comparisons revealed modest peripheral changes, including reduced mean platelet volume (MPV) and increased eosinophil counts, that persisted after false discovery rate correction. Both differences remained significant after adjustment for age and sex. Inflammatory indices from complete blood count showed poor discriminative validity and were not strongly related to the global intelligence quotient. Exploratory analyses revealed only nominal, domain-specific associations between systemic inflammation indices and particular Wechsler subtests for verbal abstraction and visuospatial organization that did not survive multiple-comparison correction. Conclusions: The findings indicate a selective peripheral hematological pattern in SLD rather than evidence of generalized systemic inflammation. The evaluated indices showed limited discriminative utility and were not independently associated with global cognitive performance after multiple-testing correction. They should not be considered screening or diagnostic biomarkers for SLD. The selective pattern of lower MPV and higher eosinophil count observed in children with SLD appears to be novel but should be considered preliminary and requires independent confirmation in larger, prospectively recruited groups.

## 1. Introduction

Specific learning disorder (SLD), termed “specific learning disability” in the Human Phenotype Ontology (HP:0001328), is a neurodevelopmental disorder characterized by persistent difficulties in learning and using academic skills that persist for at least six months despite targeted intervention, remain substantially below expectations for age, and cause clinically significant interference with academic or occupational functioning [1,2]. These difficulties must not be better explained by intellectual disability, uncorrected sensory impairment, another neurological or psychiatric condition, psychosocial adversity, inadequate proficiency in the language of instruction, or insufficient educational opportunity [1,2]. SLD may involve impairment in reading, written expression, mathematics, or more than one academic domain. The commonly used terms dyslexia, dysgraphia, and dyscalculia broadly correspond to difficulties in reading, written expression, and mathematics, respectively, although these presentations frequently overlap and should not be regarded as mutually exclusive categories [1,2]. The Diagnostic and Statistical Manual of Mental Disorders, Fifth Edition (DSM-5) estimates that SLD affects approximately 5–15% of school-age children across languages and cultures [1,2]. Within this broader international context, a two-stage epidemiological study from Türkiye reported an overall prevalence of 6.6%, with impairments in reading, mathematics, and written expression affecting 4.0%, 3.6%, and 1.8% of primary school children, respectively; 62.75% of affected children had at least one comorbid diagnosis, most commonly attention-deficit/hyperactivity disorder (54.9%) [3].

The marked clinical heterogeneity of SLD is consistent with the multiple-deficit model, in which academic difficulties emerge through interacting genetic, neurodevelopmental, cognitive, and environmental influences [4,5]. Genetic evidence supports a complex polygenic architecture rather than a single disorder-specific pathway. Several susceptibility genes, particularly *DYX1C1*, *KIAA0319*, *DCDC2*, and *ROBO1*, have historically been implicated, especially in reading-related phenotypes [6]. More recently, *PPP2R5E* was proposed as a potential additional candidate on the basis of a family-based genetic investigation [7]. These findings support biological heterogeneity but should be interpreted cautiously because individual candidate-gene associations are not uniformly replicated and do not constitute disorder-specific diagnostic markers.

Neurobiological frameworks conceptualize SLD as a disruption in the development of large-scale neural networks subserving language processing, executive control, attention, visual-perceptual binding, and information processing speed [8]. This neurodevelopmental trajectory is not independent of systemic physiology. Peripheral inflammatory states modulate the central nervous system. This neuroimmune communication is mediated through endothelial signaling, blood–brain barrier disruption, cytokine cascades and secondary glial activation [9,10,11,12,13]. Cognitive skills rely on efficient neural transmission across long-range connections. Therefore, proinflammatory mediators can interfere with synaptic wiring, oligodendrocyte maturation and white matter development, particularly during critical developmental windows [9,11,14].

Inflammatory indices derived from standard complete blood count tests provide a feasible and cost-effective method to assess peripheral immune-inflammatory balance. Researchers frequently utilize simple ratios, including the neutrophil-to-lymphocyte, monocyte-to-lymphocyte, and platelet-to-lymphocyte ratios. More complex composite metrics are also commonly employed, such as the systemic immune-inflammation index, the systemic inflammation response index, and the aggregate index of systemic inflammation [15,16,17,18]. Notably, the aggregate index of systemic inflammation uses the same mathematical formula as the pan-immune-inflammation value, which has been applied in several pediatric studies on SLD [18,19]. These blood-based metrics should not be interpreted as direct indicators of central nervous system function or as specific diagnostic tools. Instead, they serve as accessible clinical summaries that capture the general dynamics of peripheral innate immunity, lymphocyte activity, and platelet function.

In the case of SLD, the search for peripheral biomarkers has resulted in inconsistent findings at the group level in terms of immune-inflammatory and neurobiological signals. Studies using complete blood count-derived indices have reported differences in neutrophil-to-lymphocyte ratio, platelet-to-lymphocyte ratio, systemic immune-inflammation index and pan-immune-inflammation value, as well as differences in lymphocyte, monocyte and total leukocyte counts, but these results often do not replicate in independent groups [19,20,21,22]. Additional studies have investigated alternative molecular pathways and found changes in the expression of galectin-1/3, zonulin, claudin-5, cingulin, interleukin-6, matrix metalloproteinase-9 to tissue inhibitor of metalloproteinases-1 ratio, brain-derived neurotrophic factor, and agmatine-related enzymes [23,24,25,26,27,28]. Although these data expand the knowledge of the neurobiological bases of SLD, none of the markers have shown sufficient robustness for clinical or diagnostic use. Autism spectrum disorder and attention-deficit/hyperactivity disorder are also characterized by consistent documentation of peripheral inflammatory signatures. The overlap of systemic markers implies that these markers might represent a shared, transdiagnostic neurodevelopmental vulnerability rather than a pathophysiology unique to SLD [29,30,31].

Moreover, previous work on peripheral biomarkers has mostly examined case–control differences and has not focused on how these biological signals may be associated with performance on standardized tests of cognition or academic achievement. This is a major gap, especially as the Wechsler Intelligence Scale for Children—Revised (WISC-R), a mainstay in clinical practice, is designed specifically to measure a broad spectrum of cognitive abilities, including verbal abstraction, visuospatial organization, attention, and general intelligence [32,33].

Peripheral biomarkers provide useful insights into the underlying systemic physiology, but they are not sufficient to distinguish the complex cognitive phenotypes that are characteristic of neurodevelopmental conditions, and a thorough neuropsychological evaluation is necessary to confirm specific functional deficits. The integration of peripheral biological data and fine-grained cognitive profiling greatly sharpens our understanding of neurodevelopmental trajectories. An important feature is the ability to map the association between systemic biomarkers and specific cognitive domains, as peripheral inflammation may differentially affect specific neural systems involved in verbal abstraction, visuospatial organization, or processing speed. The association between routine hematological indices and standardized cognitive performance in SLD has been poorly studied, despite its biological plausibility. The aim of the present study is to fill this gap and to map the molecules-to-cognition continuum. Therefore, the objectives of the present study were: (1) to compare inflammatory indices derived from the complete blood count in a case–control setting; and (2) to evaluate the association between these peripheral markers and the individual subtest scores of the WISC-R in children with SLD.

## 2. Materials and Methods

### 2.1. Study Design and Setting

This retrospective case–control study was conducted at the child and adolescent psychiatry outpatient clinics of the University of Health Sciences, Adana City Training and Research Hospital, Adana, Türkiye. The medical records of children aged 7 to 16 years who were evaluated between 1 September 2022 and 1 September 2025, were reviewed. This specific age range was selected because it represents the primary developmental window for formal academic skill acquisition and the standard period for learning-disorder diagnosis within the Turkish educational system. The initial hospital database query for screening identified 243 unique records coded with the International Classification of Diseases, Tenth Revision codes F81 and F81.x. It is important to note that these diagnostic codes were utilized exclusively for the preliminary screening of the database. A patient was included as an SLD case only if they had a chart-documented clinical diagnosis established by the specialist child psychiatry service according to the Diagnostic and Statistical Manual of Mental Disorders, Fifth Edition criteria [1], accompanied by complete cognitive testing data. The study comprised two major analytical components. The first compared complete blood count-derived markers of inflammation alongside a variety of biochemical measures in a case–control design between children with SLD and well-child outpatients. The second examined within-group correlations between hematological indices and cognitive test results exclusively in the SLD group, aiming to map the interface between peripheral molecules and cognitive functioning.

### 2.2. Participants and Diagnostic Assessment

The SLD group consisted of 103 children diagnosed according to DSM-5 criteria following an integrated clinical evaluation. The control group comprised 102 children retrospectively identified from well-child outpatient records. Control eligibility was determined from routine medical records; controls did not undergo study-specific formal psychiatric interviews, cognitive testing, standardized academic achievement assessment, or structured neurodevelopmental or learning-disorder evaluation. Therefore, undetected subclinical learning or attentional difficulties in the control group cannot be excluded.

The exclusion criteria were applied to the SLD group through clinical assessment and to the control group through the available medical records. Participants were excluded when clinical or record-based information indicated intellectual disability, attention-deficit/hyperactivity disorder, autism spectrum disorder, anxiety, mood or psychotic disorders, neurological disease, chronic inflammatory or autoimmune disease, hematological or endocrine disorder, or malignancy. Records indicating immunomodulatory treatment or an acute inflammatory condition at the time of venipuncture were also excluded. Acute infection was defined as active clinical symptoms, documented fever, or recent antibiotic use, based on the available medical documentation. Because of the retrospective nature of the data extraction from electronic health records, several potential confounding variables were not systematically recorded. Thus, data on body mass index, allergic or atopic diseases, diet, sleep patterns, socioeconomic factors, concurrent use of medications or supplements, pubertal stage, recent vaccinations, and the season of blood sampling were either not available or too unreliable to be entered as covariates in the statistical models.

**Figure 1:**
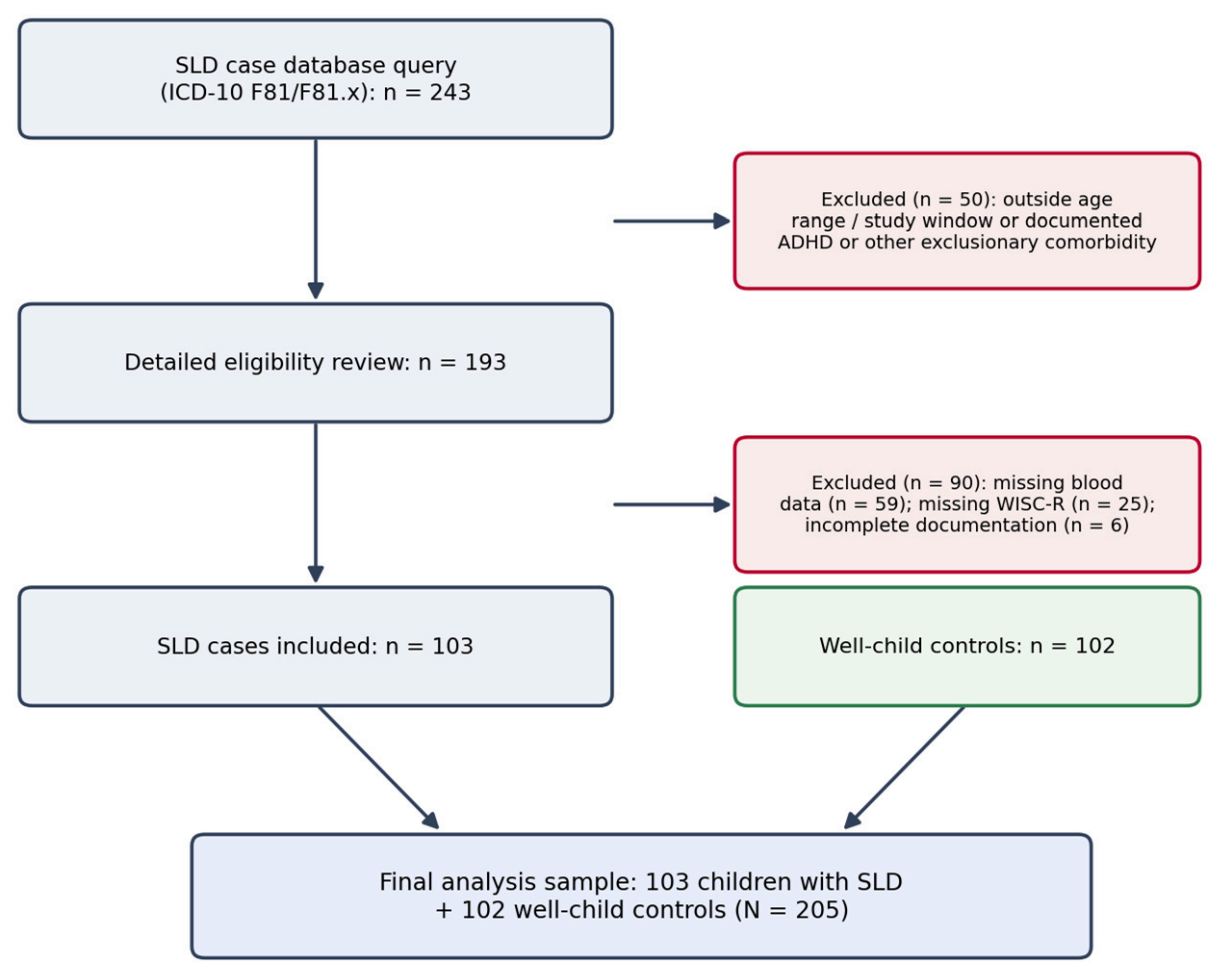
Flow diagram of participant selection. An initial search of the institutional database was conducted, identifying 243 children with a provisional diagnosis of specific learning disorder. Key exclusion criteria included falling outside the specified age or study period range, and the presence of attention-deficit/hyperactivity disorder or other exclusionary psychiatric comorbidities. Following the application of these criteria, 193 records remained for full eligibility assessment. Further exclusions were applied for missing laboratory data, absent Wechsler Intelligence Scale for Children—Revised assessments, or incomplete clinical documentation. This process resulted in a final sample of 103 children with specific learning disorder. These cases were compared with an independent control group of 102 well-child participants, for a total analytical sample of 205. Blue arrows indicate the progression of participant selection. Blue boxes represent the SLD screening and inclusion pathway, red boxes indicate exclusions, and the green box represents the well-child control group.

The selection of participants is illustrated in Figure 1. The initial search of the database returned 243 records. Following the initial screening, 50 records were removed as they fell outside the age range and the time frame of the study or had documented psychiatric or neurodevelopmental comorbidities. This led to 193 records for detailed eligibility assessment. Ninety of these were excluded due to missing laboratory data (*n* = 59), missing cognitive assessment results (*n* = 25), or incomplete clinical documentation (*n* = 6). Thus, the final sample of the study consisted of 103 children diagnosed with SLD. For comparison, 102 well-child outpatient controls meeting the record-based eligibility criteria were included through convenience sampling; they were not prospectively matched to the SLD group.

### 2.3. Cognitive Assessment

Cognitive assessment data were available only for children in the SLD group and not for controls. Children in the SLD group were assessed using the WISC-R, administered by experienced psychologists as part of the diagnostic protocol. The instrument yields Full-Scale, Verbal, and Performance Intelligence Quotients and assesses several cognitive domains. Nevertheless, the WISC-R does not provide the contemporary working-memory and processing-speed indices available in newer Wechsler editions.

The clinical diagnosis of SLD was made by a child and adolescent psychiatrist with more than 15 years of clinical experience, according to the criteria of the Diagnostic and Statistical Manual of Mental Disorders, Fifth Edition [1]. The diagnosis was based on an integrative clinical formulation, not a single metric, including detailed developmental and educational histories, structured clinical interviews, available school records, formal exclusion of intellectual disability, and a comprehensive assessment of characteristics of cognitive functioning. It should be stressed that the cognitive assessment was not used as the sole diagnostic tool for the disorder. Rather, its main purpose in the clinical workflow was to verify that the child’s intellectual functioning was above the threshold for intellectual disability and to provide a more comprehensive mapping of their cognitive abilities. Children who received a clinical diagnosis of SLD and had a Full-Scale Intelligence Quotient greater than 80 were enrolled in the case group. Because the study was retrospective, cognitive assessment data were only available for the SLD group, allowing for within-group correlational analyses to explore the associations between cognitive indices and peripheral hematological parameters. However, this retrospective extraction of data also meant that standardized academic achievement batteries and SLD subtype classifications were not systematically documented in the medical records and could not be included in the analyses.

### 2.4. Blood Sampling and Laboratory Measurements

Venous blood samples were drawn during the clinical examination for routine laboratory evaluation. The complete blood count parameters analyzed included hemoglobin, red blood cell count, white blood cell count, neutrophils, lymphocytes, monocytes, eosinophils, basophils, platelets, and mean platelet volume (MPV). The biochemical variables analyzed comprised C-reactive protein, ferritin, vitamin B12, folate, thyroid-stimulating hormone, and free thyroxine. The complete blood count was conducted on a Beckman Coulter DxH 800 hematology analyzer, biochemical parameters were measured on a Beckman Coulter AU5800 chemistry analyzer, and hormone levels were measured on a Beckman Coulter DxI 800 immunoassay system (all from Beckman Coulter Inc., Brea, CA, USA).

One extreme red blood cell observation in the SLD group (9.78 × 10^6^/µL) was identified as a transcription artifact because it was physiologically incompatible with the corresponding hemoglobin concentration. Because the de-identification protocol did not permit re-access to the source record, this single observation was excluded from red blood cell analyses (SLD *n* = 102). Retrospective records inconsistently documented fasting time, collection time, time to analysis, anticoagulant type, platelet-clumping or hemolysis flags, storage conditions, and season of sampling; these preanalytical variables could not be controlled. Complete blood count-derived inflammatory indices were calculated from absolute cell counts using the standard formulas.

### 2.5. Ethical Considerations

The study protocol was reviewed and approved by the Clinical Research Ethics Committee of the University of Health Sciences, Adana City Training and Research Hospital (Decision No: 788, 23 October 2025) and was conducted in strict accordance with the ethical standards set out in the 1964 Declaration of Helsinki. The study was retrospective, and the data were obtained from routine clinical records that were de-identified; therefore, the committee formally waived the requirement for written informed consent. Moreover, the study protocol did not include direct patient interventions, additional diagnostic procedures, or blood sampling beyond the routine care provided in daily practice.

### 2.6. Statistical Analysis

The Statistical Package for the Social Sciences software, version 26.0 (IBM Corporation, Armonk, NY, USA) was used for all statistical analyses. The distributional properties of continuous variables were examined before hypothesis testing. Normality was assessed using visual inspection of histograms and quantile–quantile plots, as well as the Shapiro–Wilk test. The basic hematological parameters, including hemoglobin, MPV, and red blood cell count, were normally distributed and met the assumptions of parametric statistical procedures. In contrast, C-reactive protein, ferritin, and the composite ratio-based inflammatory indices were right-skewed. Accordingly, between-group comparisons for these variables used non-parametric methods. Pearson correlations were retained for the primary exploratory biomarker–cognition analyses to maintain comparability with the full correlation matrix, with Spearman rank-order correlations performed as distribution-robust sensitivity analyses. The choice of descriptive statistics was based on the distributional properties of the data; normally distributed continuous data were summarized as means and standard deviations, and non-normally distributed data were summarized as medians with minimum and maximum values. Categorical variables are presented as frequencies and percentages.

Differences between groups were tested using Student’s *t*-test or Mann–Whitney U test, as appropriate; categorical variables were compared using chi-square or Fisher’s exact tests. Hedges’ g quantified unadjusted effects. Age- and sex-adjusted analyses of the principal hematological and biochemical outcomes used analysis of covariance (ANCOVA), with group as the fixed factor and age and sex as covariates; partial eta squared (ηp^2^) was reported. Because eosinophil counts were right-skewed, they were transformed using log10(x + 1) before ANCOVA. Model assumptions were evaluated from residual distributions and homogeneity-of-variance diagnostics. Sensitivity analyses excluded extreme outliers, defined as observations more than three standard deviations from the variable mean. For MPV, two observations (one SLD and one control) were excluded; for eosinophil count, five observations (three SLD and two controls) were excluded. Benjamini–Hochberg false discovery rate correction was applied within three separate test families [34]: the laboratory comparison panel, the complete blood count-derived inflammatory index panel, and the focused cognitive correlation set.

Pearson correlations were used in the primary biomarker–cognition analyses. Because ratio-based inflammatory indices were right-skewed, Spearman rank-order correlations were additionally performed as a sensitivity analysis. The focused set of 18 exploratory correlations comprised six inflammatory indices evaluated against Similarities, Picture Completion, and Full-Scale Intelligence Quotient. Similarities and Picture Completion were selected as domain-relevant subtests showing nominal signals in the broader exploratory matrix, whereas Full-Scale Intelligence Quotient served as a global cognitive benchmark. Because this focused analysis was informed partly by the observed data, it should be regarded as exploratory rather than confirmatory. The full unadjusted Pearson matrix is provided in Supplementary Table S2, and Spearman sensitivity results for all four nominal Pearson associations are provided in Supplementary Table S3.

Exploratory receiver operating characteristic (ROC) analysis was performed only to describe discriminatory performance. MPV and eosinophil count were added because they were the only laboratory markers surviving false discovery rate correction in unadjusted comparisons. No ROC result was interpreted as evidence of stand-alone diagnostic utility.

## 3. Results

### 3.1. Demographic Characteristics

A total of 205 participants were included in the final analysis, comprising 103 children with SLD and 102 well-child outpatient controls. The control group was older than the SLD group, and the sex distribution differed between the two groups, with a higher proportion of males in the SLD group than in the control group (Table 1). Because age and sex are known to influence complete blood count parameters, MPV, eosinophil counts, and endocrine markers, these demographic imbalances are treated as important interpretive limitations and potential sources of residual confounding rather than evidence that the groups were fully matched.

### 3.2. Hematological and Biochemical Parameters

**Figure 2:**
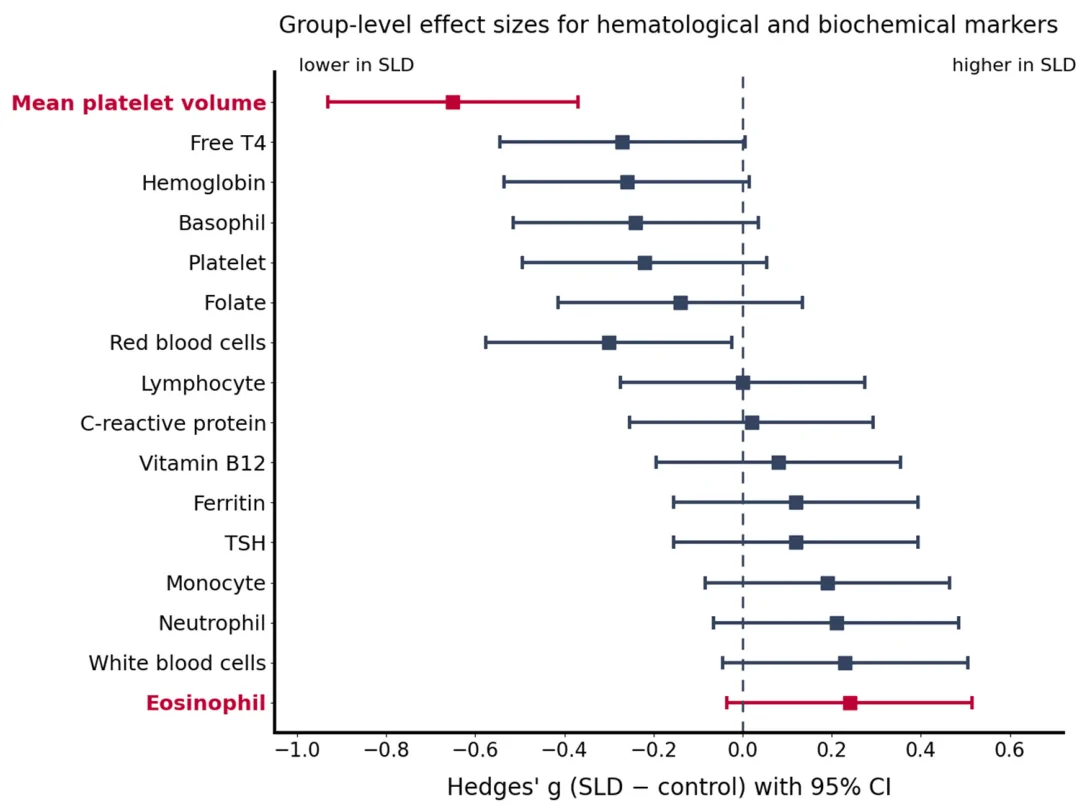
Effect sizes of hematological and biochemical markers at the group level. The mean differences (specific learning-disorder minus well-child control) between the specific learning-disorder and well-child control groups are shown as Hedges’ g values with 95% confidence intervals. Negative values indicate lower and positive values indicate higher marker levels in the specific learning disorder group. Variables that remained statistically significant after false discovery rate correction are highlighted in red. Red highlighting refers to significance after false discovery rate correction in the unadjusted between-group analyses; age- and sex-adjusted results are reported separately in Table 3. All 16 parameters listed in Table 2 are shown. Eosinophil count was compared using the Mann–Whitney U test because of right-skewness; the Hedges’ g confidence interval shown here is parametric and may therefore include zero even where the rank-based test was statistically significant. Squares represent Hedges’ g point estimates, horizontal lines represent 95% confidence intervals, and the vertical dashed line indicates the null value of zero; blue denotes results that did not survive false discovery rate correction.

The hematological and biochemical parameters are summarized in Table 2 and Figure 2. In unadjusted comparisons, MPV was lower in the SLD group than in the control group (8.95 ± 1.18 vs. 9.68 ± 1.04 fL; −7.5%; *p* < 0.001, q < 0.001; Hedges’ g = −0.65), and eosinophil count was higher (0.29 ± 0.25 vs. 0.23 ± 0.25 × 10^3^/µL; +26.1%; *p* = 0.005, q = 0.040; Hedges’ g = 0.24); both survived false discovery rate correction. Red blood cell count (4.68 ± 0.33 vs. 4.79 ± 0.39 × 10^6^/µL; −2.3%; *p* = 0.031, q = 0.152; Hedges’ g = −0.30) and free thyroxine (0.89 ± 0.15 vs. 0.93 ± 0.15 ng/dL; −4.3%; *p* = 0.038, q = 0.152; Hedges’ g = −0.27) were also lower in the SLD group, but these nominal differences did not survive false discovery rate correction. The remaining hematological and biochemical measures, including hemoglobin, white blood cell count, platelet count, neutrophil count, lymphocyte count, monocyte count, basophil count, C-reactive protein, vitamin B12, folate, ferritin, and thyroid-stimulating hormone, did not differ significantly between the groups after correction for multiple comparisons (Table 2). Percentage differences were calculated relative to the control group mean and are reported as descriptive measures of relative magnitude rather than as additional inferential statistics. After adjustment for age and sex, the differences remained significant for MPV (adjusted *p* = 0.003, ηp^2^ = 0.058) and eosinophil count (adjusted *p* = 0.021, ηp^2^ = 0.027), whereas the nominal free-thyroxine difference was attenuated (adjusted *p* = 0.085, ηp^2^ = 0.014; Table 3). In sensitivity analyses excluding observations more than three standard deviations from the mean, the MPV difference remained significant after removal of two observations (SLD *n* = 102, control *n* = 101; *p* < 0.001; Hedges’ g = −0.63), and the eosinophil-count difference remained significant after removal of five observations (SLD *n* = 100, control *n* = 100; *p* = 0.008; Hedges’ g = 0.21).

### 3.3. Complete Blood Count-Derived Inflammatory Indices

**Figure 3:**
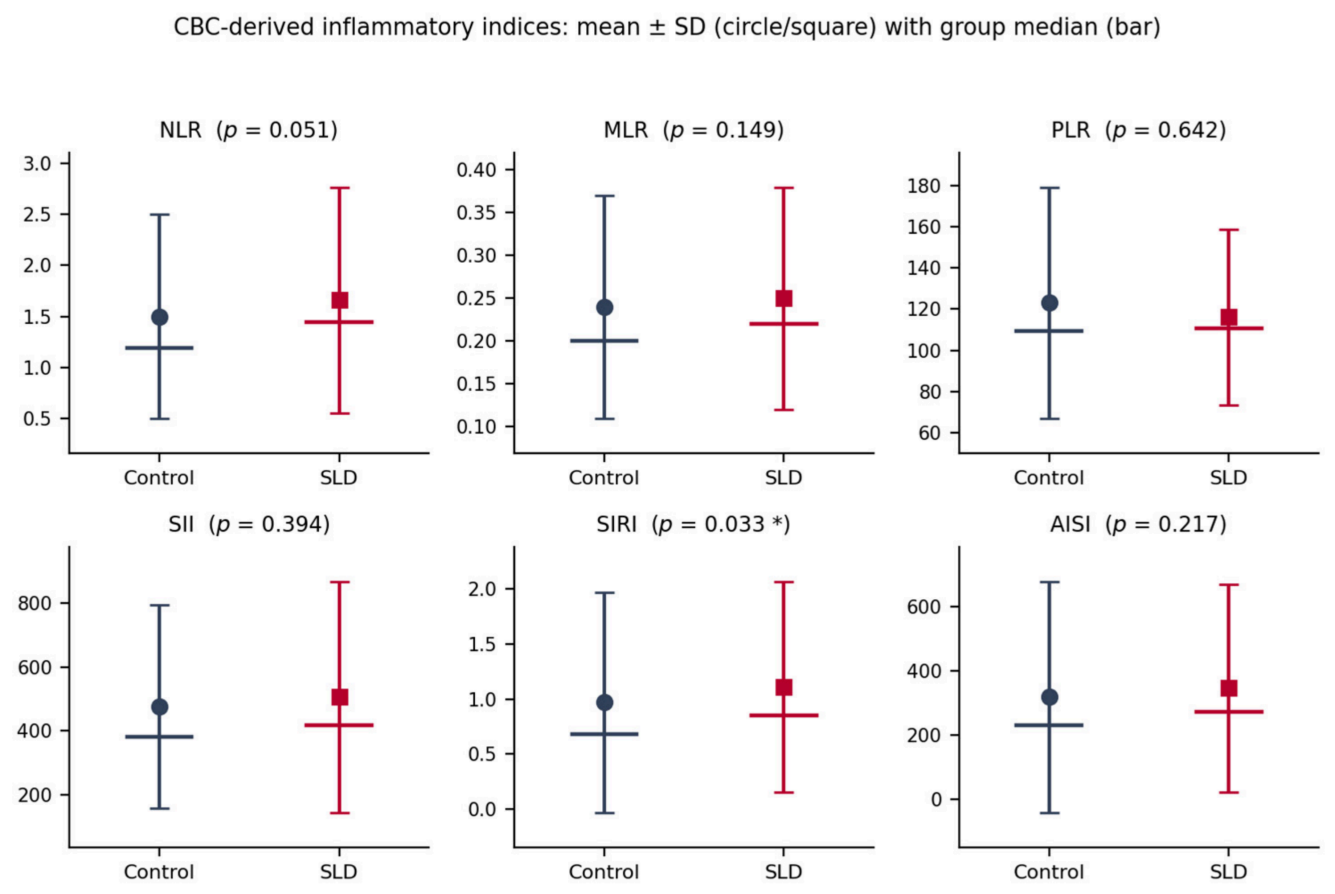
Distribution of inflammatory indices based on complete blood count in children with specific learning disorder and well-child controls. Individual symbols are group means, vertical error bars are standard deviations, and horizontal lines are group medians. The *p*-values shown in each panel are unadjusted between-group comparisons. Of the metrics evaluated, the systemic inflammation response index showed a nominal difference between the groups, while all other inflammatory indices did not reach statistical significance. Blue circles represent the control-group means, and red squares represent the specific learning-disorder group means. The asterisk indicates nominal statistical significance at *p* < 0.05; this difference did not remain statistically significant after false discovery rate correction.

Group comparisons of the complete blood count-derived inflammatory indices are presented in Table 4 and Figure 3. The systemic inflammation response index was 14.4% higher in the SLD group than in the control group (1.11 ± 0.96 vs. 0.97 ± 1.00; *p* = 0.033, q = 0.153; Hedges’ g = 0.14), but this nominal difference did not remain statistically significant after false discovery rate correction. The neutrophil-to-lymphocyte ratio was descriptively 10.7% higher in the SLD group (1.66 ± 1.11 vs. 1.50 ± 1.00; *p* = 0.051, q = 0.153; Hedges’ g = 0.15) but did not reach nominal statistical significance. The monocyte-to-lymphocyte ratio, platelet-to-lymphocyte ratio, systemic immune-inflammation index, and aggregate index of systemic inflammation also did not differ statistically between the groups after correction (Table 4).

### 3.4. Wechsler Intelligence Scale for Children—Revised Cognitive Performance and Biomarker–Cognition Associations

Within the SLD group, the selected Pearson correlations between six complete blood count-derived indices and Similarities, Picture Completion, and Full-Scale Intelligence Quotient are presented in Table 5. Four associations were nominally significant. The systemic inflammation response index (r = 0.216; *p* = 0.028; q = 0.209) and the aggregate index of systemic inflammation (r = 0.208; *p* = 0.035; q = 0.209) were positively correlated with Similarities, whereas the aggregate index of systemic inflammation (r = −0.208; *p* = 0.035; q = 0.209) and the systemic inflammation response index (r = −0.195; *p* = 0.049; q = 0.219) were negatively correlated with Picture Completion. None of these associations survived false discovery rate correction. No index was associated with Full-Scale Intelligence Quotient (all |r| ≤ 0.035; *p* ≥ 0.725). Spearman sensitivity analyses yielded the same overall conclusion. The correlations were as follows: systemic inflammation response index with Similarities (ρ = 0.201; *p* = 0.041), aggregate index of systemic inflammation with Similarities (ρ = 0.198; *p* = 0.045), systemic inflammation response index with Picture Completion (ρ = −0.189; *p* = 0.056), and aggregate index of systemic inflammation with Picture Completion (ρ = −0.194; *p* = 0.049). None survived the Benjamini–Hochberg false discovery rate correction (all q > 0.20; Supplementary Table S3).

### 3.5. Exploratory Discrimination Analyses

ROC analyses showed weak discriminatory performance (Supplementary Table S1). Among the ratio-based indices, the systemic inflammation response index had the largest area under the curve (0.600), with a cutoff of 0.775 corresponding to a sensitivity of 57 percent and a specificity of 57 percent; the areas under the curve were lower for the neutrophil-to-lymphocyte ratio (0.566), the aggregate index of systemic inflammation (0.540), the systemic immune-inflammation index (0.534), and the platelet-to-lymphocyte ratio (0.519). Additional exploratory analyses yielded an area under the curve of 0.660 for MPV and 0.580 for eosinophil count. These values do not support stand-alone screening or diagnostic utility.

## 4. Discussion

### 4.1. Main Findings

The main pattern identified from this exploratory retrospective case–control study was highly selective rather than generalized. After false discovery rate correction, only decreased MPV and increased eosinophil counts were found to be significantly different between the two groups. Conversely, the decrease in red blood cell count, the decrease in free thyroxine levels, and the rise in the systemic inflammation response index were only nominal. The groups did not significantly diverge regarding most routine hematological, biochemical, and complete blood count-derived inflammatory indices. Moreover, the composite ratio-based indices showed poor discriminative validity, and more importantly, none of the hypothesized associations between peripheral biomarkers and cognitive domains survived rigorous correction for multiple comparisons. Hence, these data do not support the presence of a peripheral diagnostic signature for SLD. Instead, the observed group-level differences in hematology are unlikely to be specific to the disorder, but instead indicative of biological heterogeneity, and will require prospective validation before any mechanistic or disorder-specific conclusions can be reached.

### 4.2. Complete Blood Count-Derived Inflammatory Indices in the Context of the SLD Literature

Previous studies of complete blood count-derived inflammatory measures in SLD have produced partially supportive but non-replicating results. Bilaç et al. reported higher neutrophil-to-lymphocyte ratio, neutrophil count, and lymphocyte count in children with SLD; however, platelet-to-lymphocyte ratio did not differ, and neutrophil-to-lymphocyte ratio was not independently associated with case status [20]. Avşar et al. found higher neutrophil-to-lymphocyte ratio, platelet-to-lymphocyte ratio, and systemic immune-inflammation index values, together with lower lymphocyte counts, and identified systemic immune-inflammation index as independently associated with SLD [21]. Mercan Işık et al. reported higher white blood cell and lymphocyte counts and a platelet-to-lymphocyte ratio difference, without a corresponding neutrophil-to-lymphocyte ratio difference [22]. More recently, Balatacı and Ay found a higher pan-immune-inflammation value, whereas the systemic immune-inflammation index and systemic inflammation response index did not differ significantly between groups [19]. Collectively, these studies support the possibility of low-grade peripheral immune variation in some samples, but they do not establish a reproducible cellular or ratio-based profile.

The present results fit this heterogeneous pattern. Neutrophil-to-lymphocyte ratio, platelet-to-lymphocyte ratio, neutrophil count, and lymphocyte count did not differ significantly between groups. The absence of a platelet-to-lymphocyte ratio difference is consistent with Bilaç et al. [20], and the absence of a neutrophil-to-lymphocyte ratio difference is closer to Mercan Işık et al. [22], but both findings contrast with Avşar et al. [21]. Among the composite indices, the systemic inflammation response index showed a nominal elevation, whereas the systemic immune-inflammation index and the aggregate index of systemic inflammation were not different. This contrasts with the systemic immune-inflammation index finding reported by Avşar et al. [21] and the pan-immune-inflammation value reported by Balatacı and Ay [19]. Because the aggregate index of systemic inflammation is mathematically equivalent to the pan-immune-inflammation value [18,19], this discrepancy cannot be attributed to terminology. Rather, the fact that different groups identify different indices suggests marker-dependent heterogeneity related to sample composition, demographic structure, exclusion criteria, comorbidity burden, nutritional or allergic background, preanalytical conditions, and distributional characteristics.

Clinical discrimination was similarly limited. In the present study, among the ratio-based indices, systemic inflammation response index produced the highest area under the ROC curve, but the area under the curve was only 0.600, with sensitivity and specificity of 57%. Avşar et al. likewise reported weak discrimination for systemic immune-inflammation index, with an area under the curve of 0.602 [21]. These closely similar values indicate that statistical group separation does not translate into clinically useful classification. Moreover, the composite indices share leukocyte and platelet components by construction [15,16,17,18]; therefore, differences among them should not be interpreted as evidence for distinct biological pathways without independent immunological validation. At present, complete blood count-derived indices are more appropriately regarded as exploratory population-level measures than as individual diagnostic biomarkers.

### 4.3. Mean Platelet Volume: A Notable but Preanalytically Sensitive Finding

The most notable group-level effect observed in the current study was a reduction in MPV, which remained statistically significant even after correction for false discovery rate. Although leukocyte- and platelet-based ratios have dominated the SLD literature [19,20,21,22], MPV has been comparatively underexplored in this context, despite prior investigation of its role as a peripheral inflammatory marker in pediatric attention-deficit/hyperactivity disorder [35]. In our group, the absence of a significant difference in absolute platelet counts indicates that this finding reflects platelet size rather than the total number of platelets. However, MPV is an indirect and non-specific surrogate marker; thus, the present data do not allow us to conclude whether the observed decrease is due to increased platelet activation, increased turnover, an alternative physiological mechanism, or simply pre-analytical measurement conditions.

This interpretative limitation is particularly relevant because MPV is highly sensitive to pre-analytical variables, including the type of anticoagulant, the time interval between venipuncture and analysis, storage temperature, specific analyzer characteristics, and in vitro platelet swelling or clumping [36]. Due to the retrospective design of this study, these methodological parameters were neither standardized nor routinely documented in the medical records. Thus, the decreased MPV observed in this group should be considered only a statistically significant finding at the group level, not conclusive evidence for a separate platelet-mediated pathophysiological subtype of SLD. Prospective studies are needed, using strictly standardized blood collection protocols and prespecified guidelines for the choice of anticoagulant, processing times, storage conditions, and analyzer calibration, to validate this finding.

### 4.4. Secondary Immune-Endocrine Findings

The higher eosinophil counts also remained significant after false discovery rate correction, but their effect size was small, and their interpretation is highly sensitive to confounders. Eosinophils participate in type 2 immunity and in tissue and immune homeostasis [37,38]; however, peripheral eosinophil counts are strongly influenced by allergic and atopic disease, parasitic exposure, medications, recent immune activation, and seasonal variation [37]. Eosinophil count was not identified as a significant between-group marker in the recent Balatacı and Ay group [19], and it has not emerged as a consistent feature across the SLD literature [19,20,21,22]. Because allergic status, atopic disease, environmental exposure, medication use, and season of sampling were not systematically available, the present finding is best interpreted as a nonspecific peripheral immune difference rather than a stable component of SLD biology.

Likewise, biochemical analyses did not show a consistent metabolic or endocrine pattern. There were no significant differences in the concentrations of vitamin B12, folate, ferritin, C-reactive protein, and thyroid-stimulating hormone between the groups. Consistent with the present results, Mercan Işık et al. showed no consistent changes in vitamin B12, folate, thyroid-stimulating hormone, or thyroxine among different severities of SLD [22]. In addition, Balatacı and Ay reported decreased vitamin B12 levels but no significant between-group differences in C-reactive protein, folate, thyroid-stimulating hormone, or free thyroxine [19]. In the present study, free thyroxine was lower only at the uncorrected level and did not survive false discovery rate correction. Although thyroid hormones are essential for cortical maturation and myelination [39], and inflammatory signaling can alter peripheral thyroid hormone metabolism [40], the unchanged thyroid-stimulating hormone level and absence of longitudinal endocrine measurements preclude an interpretation of specific thyroid-axis dysfunction. The free thyroxine finding should therefore remain hypothesis-generating.

### 4.5. Cognitive Findings and the Molecules-to-Cognition Question

Within the SLD group, systemic inflammation response index and aggregate index of systemic inflammation showed nominal associations with the Similarities and Picture Completion subtests, but the associations were weak, occurred in different directions across domains, and did not survive false discovery rate correction. No complete blood count-derived index was associated with Full-Scale Intelligence Quotient. This pattern argues against interpreting these peripheral indices as markers of generalized cognitive impairment and provides no evidence for a direct linear molecules-to-cognition pathway.

In a related investigation, Balatacı and Ay found stronger relationships between cell-based inflammatory composites, particularly the pan-immune-inflammation value, and the working-memory and processing speed domains of the Wechsler Intelligence Scale for Children—Fourth Edition [19]. These analyses, however, were exploratory and not corrected for multiple comparisons, but they suggested the intriguing possibility that neuroimmune associations in SLD are domain-specific rather than a reflection of global cognitive functioning. The methodological differences between the studies are also highly relevant; unlike the fourth edition, the present study used the WISC-R, which does not produce separate working-memory and processing speed indices [32,33]. As processing speed is inherently related to white matter development and the efficiency of neural networks [41,42], these specific cognitive domains are biologically plausible targets for future neuroimmunology research. However, the present dataset does not allow us to elucidate underlying white matter or neural circuitry mechanisms. The divergent findings between the two studies may also be explained by variations in sample severity, the specific cognitive instruments used, and differing statistical frameworks. Future research should attempt to combine contemporary versions of the Wechsler scales with standardized measures of academic achievement and rigorous correction for multiple testing.

### 4.6. Alternative Explanations and Disorder Specificity

There are several alternative explanations that limit a disorder-specific interpretation. Indices derived from complete blood counts and immune-endocrine measures are non-specific and can be confounded by recent infection, allergic or atopic disease, body composition, sleep, diet, psychological stress, socio-economic conditions, pubertal development, medication or supplement exposure, and season of sampling. Most of these variables were unavailable or incompletely recorded. Their effects are of direct relevance to the eosinophil, MPV, and thyroid findings, and may influence between-study variations in ratio-based indices.

SLD is itself clinically heterogeneous and frequently overlaps with other psychiatric and neurodevelopmental conditions [2,4]. In a Turkish epidemiological study, 62.75% of children with SLD had at least one comorbid diagnosis, and attention-deficit/hyperactivity disorder was the most frequent comorbidity, affecting 54.9% [3]. Although documented major psychiatric and neurodevelopmental comorbidities were excluded in the present study, subthreshold attentional, emotional, behavioral, and sleep-related symptoms could not be fully assessed retrospectively. Peripheral inflammatory alterations have also been reported in autism spectrum disorder and attention-deficit/hyperactivity disorder [29,30,31]. Therefore, any reproducible signal from a complete blood count might suggest transdiagnostic vulnerability for neurodevelopment or unmeasured background health variation, rather than a specific mechanism for SLD. Clinical comparison groups are needed to test specificity.

### 4.7. Diagnostic and Clinical Interpretation

Previous studies have indicated the potential relevance of complete blood count-derived indices as biomarkers in SLD [19,20,21]. However, there is currently insufficient evidence to support their use for screening, diagnosis, monitoring, prognosis or treatment selection. In the present study none of the ratio-based indices survived false discovery rate correction. The maximal area under the curve was 0.66 for MPV, whereas eosinophil count yielded 0.58 and the cognitive associations did not pass multiple testing correction. These findings highlight that statistical significance, if any, is not a substitute for clinical utility. Routine laboratory tests may be useful in identifying general medical conditions or nutritional or endocrine factors contributing to academic problems but are not a substitute for comprehensive clinical, developmental, educational, psychiatric, and neuropsychological assessments.

### 4.8. Strengths and Limitations

The study has a number of strengths. It had a relatively large clinical sample of SLD with chart-documented DSM-5-based diagnosis and available cognitive testing, used an independent well-child outpatient comparison group, evaluated both routine laboratory measures and several derived indices, reported effect sizes, and applied false discovery rate correction within separate families of tests. Emphasis on corrected results, weak discrimination and negative cognitive findings reduces the likelihood of over-stating exploratory associations.

There are several important limitations. The retrospective, single-center design precludes causal inference. Controls selected from well-child records did not undergo structured psychiatric, cognitive, academic, or neurodevelopmental assessment; unrecognized subclinical learning or attentional difficulties cannot be excluded. Because controls were identified retrospectively from routine records by convenience sampling rather than prospectively matched to the specific learning-disorder group, selection bias cannot be excluded. Although principal outcomes were adjusted for age and sex, residual confounding remains possible. Allergic and atopic status, medication use, recent infections not captured in records, and sampling season were unavailable, which is particularly important for eosinophil count. Blood collection and processing conditions, including anticoagulant type and time to analysis, were not standardized, which is especially problematic for MPV. Cognitive data were available only for the SLD group; the WISC-R lacks contemporary working-memory and processing-speed indices, and standardized academic achievement testing and systematic SLD subtype classification were unavailable.

### 4.9. Future Directions

Future studies should be prospective, multicentric, with age- and sex-matched or adequately adjusted comparison groups, and standardized blood collection, processing, and analyzer procedures. Repeated sampling should consider fasting status, time of day, season, infection history, allergic and atopic disease, body mass index, pubertal development, diet, sleep, socioeconomic conditions and medication or supplement exposure. Direct measures of inflammation and immune function should be combined with measures of nutrition and endocrine function. Comparison groups that are clinically informative should include isolated SLD, SLD with attention-deficit/hyperactivity disorder, attention-deficit/hyperactivity disorder without SLD, autism spectrum disorder, developmental language disorder, and mixed neurodevelopmental presentations. Further studies using modern cognitive batteries, standardized academic achievement tests and analyses by subtype are needed to assess whether any peripheral signal is related to specific cognitive or academic domains, rather than to case–control status only.

## 5. Conclusions

We found a selective peripheral hematologic pattern rather than strong evidence of generalized systemic inflammation in children with SLD in this exploratory retrospective case–control study. Lower MPV and higher eosinophil count were statistically significant after false discovery rate correction and remained significant after adjustment for age and sex, while lower red blood cell count, lower free thyroxine, and higher systemic inflammation response index were nominal findings only. Most hematological, biochemical, and ratio-based inflammatory markers were similar between groups. Complete blood count indices showed weak discrimination and no corrected association with Full-Scale Intelligence Quotient or selected cognitive domains. These measures should not be used as screening or diagnostic biomarkers for SLD. These findings are best considered hypothesis-generating observations that need to be prospectively replicated with demographic adjustment, standardized laboratory procedures, detailed assessment of allergic and environmental confounders, direct immunological measurement, clinical comparison groups, and current cognitive and academic assessment.

## Figures and Tables

**Table 1:** Demographic characteristics of the study groups.

Variable	Control (*n* = 102)	Specific Learning Disorder (*n* = 103)	*p*-Value
Age, years (mean ± standard deviation)	10.74 ± 3.74	9.32 ± 2.32	0.001
Female sex, *n* (%)	54 (52.9)	38 (36.9)	0.030
Male sex, *n* (%)	48 (47.1)	65 (63.1)	—

**Note.** Values are presented as mean ± standard deviation or *n* (%). Age was compared using Welch’s independent-samples *t*-test, and sex distribution was compared using the chi-square test. The *p*-value for sex refers to the overall sex distribution. The groups differed in both age (*p* = 0.001) and sex distribution (*p* = 0.030); these demographic imbalances are interpreted as important limitations and potential sources of residual confounding.

**Table 2:** Basic hematological and biochemical parameters in the control and specific learning-disorder groups.

Parameter	Control	Specific Learning Disorder	*p*-Value	q-Value	Hedges’ g
Hemoglobin (g/dL)	12.96 ± 1.22; 12.85 (10.20–17.10)	12.66 ± 1.11; 12.70 (7.11–14.70)	0.067	0.195	−0.26
Red blood cell count (10^6^/µL)	4.79 ± 0.39; 4.77 (3.29–5.74)	4.68 ± 0.33; 4.66 (4.00–5.68)	0.031	0.152	−0.30
White blood cell count (10^3^/µL)	7.68 ± 2.38; 7.38 (3.44–18.61)	8.25 ± 2.51; 8.00 (4.10–17.30)	0.104	0.238	0.23
Platelet count (10^3^/µL)	325.71 ± 79.55; 318.00 (147.00–578.00)	307.74 ± 81.57; 306.00 (151.00–699.00)	0.131	0.244	−0.22
Neutrophil count (10^3^/µL)	3.84 ± 1.86; 3.50 (1.37–13.68)	4.23 ± 1.81; 3.90 (1.60–10.60)	0.073	0.195	0.21
Lymphocyte count (10^3^/µL)	2.94 ± 1.02; 2.77 (0.50–5.92)	2.94 ± 1.22; 2.76 (0.50–8.26)	0.531	0.607	0.00
Monocyte count (10^3^/µL)	0.61 ± 0.21; 0.60 (0.31–1.81)	0.65 ± 0.22; 0.60 (0.30–1.40)	0.137	0.244	0.19
Eosinophil count (10^3^/µL)	0.23 ± 0.25; 0.17 (0.00–1.60)	0.29 ± 0.25; 0.20 (0.00–1.57)	0.005	0.040	0.24
Basophil count (10^3^/µL)	0.05 ± 0.03; 0.04 (0.00–0.10)	0.04 ± 0.05; 0.03 (0.00–0.20)	0.198	0.317	−0.24
Mean platelet volume (fL)	9.68 ± 1.04; 9.80 (6.40–12.00)	8.95 ± 1.18; 9.00 (6.50–11.30)	<0.001	<0.001	−0.65
C-reactive protein (mg/L)	2.86 ± 3.46; 2.00 (0.20–24.90)	2.94 ± 3.31; 2.10 (0.02–21.00)	0.702	0.749	0.02
Vitamin B12 (pg/mL)	253.12 ± 125.17; 223.50 (72.00–913.00)	263.24 ± 118.06; 232.00 (92.00–641.00)	0.475	0.585	0.08
Folate (ng/mL)	10.07 ± 3.70; 9.70 (3.68–20.00)	9.54 ± 3.61; 8.98 (3.27–20.43)	0.302	0.416	−0.14
Ferritin (ng/mL)	19.97 ± 12.43; 18.15 (2.60–60.80)	21.41 ± 12.01; 18.60 (3.90–61.99)	0.312	0.416	0.12
Free thyroxine (ng/dL)	0.93 ± 0.15; 0.90 (0.67–1.30)	0.89 ± 0.15; 0.86 (0.61–1.54)	0.038	0.152	−0.27
Thyroid-stimulating hormone (µIU/mL)	2.32 ± 0.92; 2.14 (0.74–5.60)	2.45 ± 1.28; 2.19 (0.34–6.80)	0.942	0.942	0.12

**Note.** Values are mean ± standard deviation; median (minimum–maximum). The *p*-values are nominal group-comparison values (Student’s *t*-test or Mann–Whitney U test); the *q*-values are Benjamini–Hochberg false discovery rate-corrected within the laboratory-comparison family; Hedges’ *g* represents the effect size (SLD minus control). The false discovery rate-surviving laboratory findings were mean platelet volume and eosinophil count. For red blood cell count, the specific learning-disorder group comprises 102 participants because one observation was excluded as a transcription artifact (Section 2.4).

**Table 3:** Age- and sex-adjusted analyses for selected hematological and biochemical parameters.

Parameter	Unadjusted *p*-Value	Adjusted *p*-Value *	Partial Eta Squared (ηp^2^)
Mean platelet volume (fL)	<0.001	0.003	0.058
Eosinophil count (10^3^/µL) †	0.005	0.021	0.027
Free thyroxine (ng/dL)	0.038	0.085	0.014

* Adjusted *p*-values were calculated using analysis of covariance with age and sex entered as covariates. † Eosinophil counts were transformed using log10(x + 1) before ANCOVA because of right-skewness.

**Table 4:** Comparison of complete blood count-derived inflammatory indices between the control and specific learning-disorder groups.

Index	Control	Specific Learning Disorder	*p*-Value	q-Value	Hedges’ g
Neutrophil-to-lymphocyte ratio	1.50 ± 1.00; 1.19 (0.51–7.00)	1.66 ± 1.11; 1.44 (0.26–7.57)	0.051	0.153	0.15
Monocyte-to-lymphocyte ratio	0.24 ± 0.13; 0.20 (0.11–1.00)	0.25 ± 0.13; 0.22 (0.08–1.00)	0.149	0.298	0.09
Platelet-to-lymphocyte ratio	123.16 ± 56.08; 109.34 (55.74–520.00)	116.13 ± 42.70; 110.83 (42.59–302.00)	0.642	0.642	−0.14
Systemic immune-inflammation index	477.15 ± 319.37; 381.71 (138.67–1820.00)	506.80 ± 363.13; 417.77 (117.53–2642.43)	0.394	0.473	0.09
Systemic inflammation response index	0.97 ± 1.00; 0.68 (0.18–8.15)	1.11 ± 0.96; 0.85 (0.17–6.81)	0.033	0.153	0.14
Aggregate index of systemic inflammation	319.22 ± 358.95; 230.74 (54.87–2736.72)	346.17 ± 322.55; 271.55 (47.01–2378.19)	0.217	0.326	0.08

**Note.** Values are mean ± standard deviation; median (minimum–maximum). None of the complete blood count-derived indices survived false discovery rate correction; the systemic inflammation response index showed only a nominal group difference. The *q*-value is the Benjamini–Hochberg false discovery rate-adjusted *p*-value.

**Table 5:** Selected exploratory correlations between complete blood count-derived inflammatory indices and Wechsler Intelligence Scale for Children—Revised measures in the specific learning-disorder group (*n* = 103).

Index	Similarities	Picture Completion	Full-Scale Intelligence Quotient
Neutrophil-to-lymphocyte ratio	r = 0.142; *p* = 0.154; q = 0.316	r = −0.140; *p* = 0.158; q = 0.316	r = 0.005; *p* = 0.963; q = 0.992
Monocyte-to-lymphocyte ratio	r = 0.170; *p* = 0.087; q = 0.261	r = −0.109; *p* = 0.272; q = 0.490	r = 0.001; *p* = 0.992; q = 0.992
Platelet-to-lymphocyte ratio	r = 0.068; *p* = 0.494; q = 0.740	r = −0.082; *p* = 0.412; q = 0.675	r = −0.035; *p* = 0.725; q = 0.992
Systemic immune-inflammation index	r = 0.144; *p* = 0.147; q = 0.316	r = −0.172; *p* = 0.082; q = 0.261	r = −0.014; *p* = 0.889; q = 0.992
Systemic inflammation response index	r = 0.216; *p* = 0.028; q = 0.209	r = −0.195; *p* = 0.049; q = 0.219	r = 0.028; *p* = 0.776; q = 0.992
Aggregate index of systemic inflammation	r = 0.208; *p* = 0.035; q = 0.209	r = −0.208; *p* = 0.035; q = 0.209	r = 0.015; *p* = 0.878; q = 0.992

**Note.** Pearson correlation coefficients are shown for the nominally notable subtests and Full-Scale Intelligence Quotient; the complete unadjusted correlation matrix is provided in Supplementary Table S2. Benjamini–Hochberg false discovery rate correction was applied across the 18 focused exploratory correlations in this table; none remained statistically significant after correction. The q-value is the false discovery rate-adjusted *p*-value.

## Data Availability

The data analyzed during the current study are not publicly available because they are derived from clinical records, but they may be made available from the corresponding author upon reasonable request and with institutional approval.

## Supplementary Materials

The following supporting information can be downloaded at https://www.mdpi.com/article/10.3390/brainsci16080776/s1, Table S1: Exploratory receiver operating characteristic analyses of complete blood count-derived inflammatory indices, mean platelet volume, and eosinophil count; Table S2: Full unadjusted Pearson correlation matrix between complete blood count-derived inflammatory indices and Wechsler Intelligence Scale for Children-Revised measures in the specific learning disorder group (n = 103). Each cell shows the correlation coefficient with the unadjusted *p*-value in parentheses; Table S3: Selected Spearman rank-order sensitivity analyses for nominal Pearson biomarker–cognition associations in the specific learning disorder group (n = 103).
