# Supplementary material for: Peripheral Hematological and Immune-Endocrine Markers in Children with Specific Learning Disorder: An Exploratory Retrospective Case–Control Study
Source: Brain Sci. 2026 Jul 23;16(8):776. doi: 10.3390/brainsci16080776 (PMC13510338; doi:10.3390/brainsci16080776)
Supplement: Supplementary file 1 [file brainsci-16-00776-s001.zip › brainsci-4417612-supplementary.pdf]

Table S1. Exploratory receiver operating characteristic analyses of complete blood count-derived inflammatory indices, mean platelet volume, and eosinophil count.

Index	Area under the curve	Cutoff	Sensitivity (%)	Specificity (%)
Neutrophil-to-lymphocyte ratio	0.566	Not applicable	Not applicable	Not applicable
Platelet-to-lymphocyte ratio	0.519	Not applicable	Not applicable	Not applicable
Systemic immune-inflammation index	0.534	Not applicable	Not applicable	Not applicable
Systemic inflammation response index	0.600	0.775	57	57
Aggregate index of systemic inflammation	0.540	Not applicable	Not applicable	Not applicable
Mean platelet volume	0.660	Not applicable	Not applicable	Not applicable
Eosinophil count	0.580	Not applicable	Not applicable	Not applicable

Note. ROC analyses were exploratory and were not intended to establish diagnostic thresholds. Mean platelet volume had the largest area under the curve (0.660), followed by systemic inflammation response index (0.600) and eosinophil count (0.580). These values indicate weak discrimination.

Table S2. Full unadjusted Pearson correlation matrix between complete blood count-derived inflammatory indices and Wechsler Intelligence Scale for Children-Revised measures in the specific learning disorder group (n = 103). Each cell shows the correlation coefficient with the unadjusted p-value in parentheses.

Measure	NLR	MLR	PLR	SII	SIRI	AISI
Verbal Intelligence Quotient	0.095 (0.342)	0.131 (0.186)	0.028 (0.778)	0.086 (0.386)	0.184 (0.063)	0.164 (0.099)
Information	0.016 (0.870)	0.023 (0.815)	−0.062 (0.532)	−0.010 (0.923)	0.066 (0.508)	0.048 (0.629)
Similarities	0.142 (0.154)	0.170 (0.087)	0.068 (0.494)	0.144 (0.147)	0.216 (0.028)	0.208 (0.035)
Arithmetic	0.101 (0.310)	0.100 (0.314)	0.022 (0.828)	0.086 (0.386)	0.184 (0.062)	0.159 (0.110)
Vocabulary	0.057 (0.569)	0.071 (0.479)	0.009 (0.925)	0.064 (0.519)	0.116 (0.243)	0.105 (0.293)
Comprehension	0.019 (0.851)	0.109 (0.272)	−0.002 (0.985)	0.019 (0.852)	0.130 (0.191)	0.115 (0.248)
Performance Intelligence Quotient	−0.067 (0.504)	−0.105 (0.291)	−0.079 (0.426)	−0.092 (0.356)	−0.104 (0.294)	−0.112 (0.262)
Picture Completion	−0.140 (0.158)	−0.109 (0.272)	−0.082 (0.412)	−0.172 (0.082)	−0.195 (0.049)	−0.208 (0.035)
Picture Arrangement	0.030 (0.760)	−0.012 (0.906)	−0.040 (0.689)	0.024 (0.811)	0.057 (0.567)	0.055 (0.583)
Block Design	−0.068 (0.497)	−0.141 (0.154)	−0.098 (0.327)	−0.079 (0.427)	−0.106 (0.284)	−0.095 (0.338)
Object Assembly	−0.019 (0.850)	−0.075 (0.450)	−0.021 (0.835)	−0.054 (0.585)	−0.091 (0.360)	−0.113 (0.258)
Coding	−0.065 (0.511)	−0.101 (0.312)	−0.078 (0.432)	−0.087 (0.383)	−0.099 (0.320)	−0.105 (0.291)
Full Scale Intelligence Quotient	0.005 (0.963)	0.001 (0.992)	−0.035 (0.725)	−0.014 (0.889)	0.028 (0.776)	0.015 (0.878)

Note. *Values are Pearson correlation coefficients with unadjusted p-values in parentheses. NLR = neutrophil-to-lymphocyte ratio; MLR = monocyte-to-lymphocyte ratio; PLR = platelet-to-lymphocyte ratio; SII = systemic immune-inflammation index; SIRI = systemic inflammation response index; AISI = aggregate index of systemic inflammation. Column headings use these abbreviations solely to accommodate the table width.*

Table S3. Selected Spearman rank-order sensitivity analyses for nominal Pearson biomarker–cognition associations in the specific learning disorder group (n = 103).

Inflammatory index	Cognitive measure	Spearman's ϱ	p-value	FDR interpretation
Systemic inflammation response index	Similarities	0.201	0.041	q > 0.20; not significant
Aggregate index of systemic inflammation	Similarities	0.198	0.045	q > 0.20; not significant
Systemic inflammation response index	Picture Completion	−0.189	0.056	q > 0.20; not significant
Aggregate index of systemic inflammation	Picture Completion	−0.194	0.049	q > 0.20; not significant

Note. These non-parametric sensitivity analyses correspond to the nominal Pearson signals highlighted in the main text. None of these associations survived Benjamini–Hochberg false discovery rate correction.
